# Symptoms of the suicide crisis syndrome and associated risk factors in an acute psychiatric population, a cross-sectional study

**DOI:** 10.1192/j.eurpsy.2025.10030

**Published:** 2025-06-13

**Authors:** Linde Melby, Karina Høyen, Astrid Prestmo, Arne Vaaler, Tuva Kvithyld, Igor Galynker, Fredrik Walby, Mette Langaas, Terje Torgersen

**Affiliations:** 1 Department of Mental Healthcare, St. Olav’s Hospital, Trondheim University Hospital, Trondheim, Norway; 2 Department of Mental Health, Norwegian University of Science and Technology, Trondheim, Norway; 3 Department of Psychiatry, Mount Sinai Beth Israel, New York, NY, USA; 4 National Centre for Suicide Research and Prevention (NSSF), Oslo University Hospital, Oslo, Norway; 5 Department of Mathematical Sciences, Norwegian University of Science and Technology (NTNU), Trondheim, Norway

**Keywords:** agitation, risk factors, suicidal behavior, suicide crisis syndrome, warning signs

## Abstract

**Background:**

The focus of suicide research changes from traditional risk factors to acute warning signs. Patient self-reported suicidal ideation (SI) is not a reliable measure of acute suicide risk. Presuicidal syndromes such as suicide crisis syndrome (SCS) attempt to describe measurable syndromes based on warning signs other than SI.

**Methods:**

Seven hundred and ten acutely admitted patients were included in the study. Identification of symptoms describing the five components of SCS was done by performing a retrospective text analysis of the patient records (electronic medical records). Patients were grouped according to high or low level of SCS symptoms. We performed statistical tests for group differences in demographics, traditional risk factors, and clinical variables, including agitation assessed by the Positive and Negative Symptom Scale-Excited Component (PANSS-EC).

**Results:**

Seventy-two patients had high levels of SCS symptoms. They reported less SI the last month before admission; suicidality was less relevant for referral, the intake suicide assessment more often concluded with high suicide risk, they were more often referred and admitted involuntarily, and they had higher total scores on PANSS-EC.

**Conclusion:**

The individual SCS symptoms may provide useful information in the evaluation of acute suicide risk at intake. A high level of SCS symptoms suggests more severe conditions. The lower reports among high-level than low-level SCS patients of self-reported SI last month before admission, shows the limitation of using SI as a warning sign. The association between the level of SCS symptoms and PANSS-EC total score suggests that agitation could give valuable additional information for suicide risk assessments.

## Introduction

There is a growing interest in identifying presuicidal syndromes consisting of symptomatic and behavioral signs of acute and high risk of suicide, not depending on self-report of suicidal ideation (SI) [1, 2]. A number of studies have shown that the patients’ self-report of SI, or absence of SI, is not a reliable measure to predict acute suicide risk [1, 3, 4]. Many individuals deny SI when last asked before dying by suicide [3, 5]. Deisenhammer et al. [6] found that SI could appear as close in time as minutes preceding a suicide attempt, and a fluctuating course has been described [7]. Therefore, it is important to identify measures or warning signs for acute suicidality, not relying on self-report of SI alone [1]. The concept of presuicidal syndrome was introduced by Ringel in 1958 [8], describing a mental state characterized by constriction, inhibited aggression to the self, and SI. Newer versions of presuicidal syndromes are the suicide crisis syndrome (SCS) proposed by Galynker [9], and the Acute Suicidal Affective Disturbance (ASAD) proposed by Rogers et al. [10]. Both the SCS and the ASAD describe a mental state characterized by overarousal, cognitive rigidity, social withdrawal, and perception of hopeless intractability. The most important difference is that suicidal intent is a key feature in the ASAD, while in the SCS, it is not a required criterion but might be present.

The present concept of SCS was introduced in 2016 and is now a proposed suicide-specific diagnosis [11–14]. There is, however, an ongoing discussion about the pros and cons of this diagnosis [15–17]. Arguments supporting the SCS as a diagnosis are several. The SCS provides a structured, systematic method to assess imminent suicide risk, and represents state-based risk factors and diagnostic criteria that can be integrated into patient psychoeducation [17]. The SCS consists of five components grouped into the A- and B-criteria. The A-criterion is a feeling of entrapment or frantic hopelessness. The B-criterion requires at least one symptom from all four subgroups: affective dysregulation, disturbance in arousal, loss of cognitive control, and social withdrawal. To measure the construct of SCS, the 49-item questionnaire Suicide Crisis Inventory (SCI) was developed [18]. Later, a revised 61-item version (SCI-2) arose [19]. Several studies have shown the usefulness of SCI and SCI-2 in predicting suicidal behavior (SB) shortly after discharge from psychiatric wards [18–20]. Each of its criteria is validated and has predictive value for SB [12], but it is not yet established an association between the SCS and completed suicides. Melzer et al. [14] recently summarized the current research on SCS and found that most studies so far were conducted in the United States, using small samples, and aimed to describe the associations between the proposed criteria and SB. As far as we know, there is only one previous study, performed by Rogers et al. [21], which aimed to explore the occurrence of SCS symptoms, and that study was not conducted in a patient population.

The SCS B-criteria, except social withdrawal, have similarities with the condition of agitation, described as a sense of restlessness or painful mental arousal accompanied by motoric hyperactivity [22, 23]. The condition is identified by skilled healthcare staff and indicates acute psychiatric illness [24, 25]. Agitation has been implicated as an acute risk factor or warning sign for SB by several authors [1, 3, 26], and a meta-analysis has shown a moderate association between agitation and SB [23]. The assessment of agitation can be measured by several instruments [22, 27], and a frequently used measure in acute and emergency settings is the Positive and Negative Symptom Scale Excited Component (PANSS-EC) [28].

A previous study from Prestmo et al. [29] has shown that the incidence of recent SI and SB is high in acutely admitted psychiatric patients in Norway and that a certain level of suicidality is related to 70% of the admissions. There is reason to assume that the incidence of SCS symptoms is high in this population. In this study, we will explore symptoms of SCS in a large sample of acutely admitted psychiatric patients. The first aim is to compare patients with high levels of SCS symptoms to patients with low levels of SCS symptoms, in relation to clinical variables like recent SI and SB, diagnosis, and rate of involuntary admission. The second aim is to assess the association between SCS symptoms and agitation, as measured by the PANSS-EC.

## Methods

### Sample and setting

The study had a quantitative cross-sectional, one-site design. The acutely admitted inpatients that comprise the sample were included in two consecutive clinical research projects at the Department of Acute Psychiatry, St. Olav’s University Hospital, Trondheim, Norway. The department has a catchment area of 300,000 inhabitants in both rural and urban areas and is the only acute department in the area. The Acute Agitation study (AA study) [30] included patients from 2011 to 2012, and the Genetic and Affective Prediction study (GAP study) [5] was conducted from 2016 to 2017.

All patients who spoke English or Norwegian and were able to provide informed consent were offered voluntary study participation. In the AA sample, 380 of 760 (50%) eligible patients consented to participate in the study. In the GAP sample, 347 of 1231 (28%) eligible patients’ consented. Seventeen patients appeared in both studies and were removed from the GAP sample. The two samples were pooled and consisted of 710 unique individuals. For further information, see section “Assessment of sample pooling” and Table A1 in the Appendix.

At admission, the physician on duty performed a regular physical and mental examination of the patient, including a standardized suicide risk assessment based on national guidelines for suicide prevention [31] and local procedures [30]. A psychiatrist or specialist in clinical psychology assessed the patients within 24 hours of admission and performed a second suicide risk assessment. The clinicians’ suicide risk assessments were documented in the patients’ electronic medical records (EMR). Most of the data for the present study were collected from the EMR by members of the research group.

### Evaluation of the sample pooling

To discover possible differences between the recorded explanatory variables in the AA versus GAP sample, we fitted univariate logistic regressions to give estimated odds ratio (OR), 95% confidence interval (CI) for the OR, and p-value for testing if the OR was equal to 1, and false discovery rate (FDR)-adjusted p-values using the Benjamini–Hochberg procedure [32]. See Table A1 in the Appendix.

### Measures: Outcome variable

The EMR from the admission was retrospectively text-analyzed for SCS symptoms by PhD candidates and research assistants affiliated with the Acute Psychiatric Department. A description of the words and terms used in the text analysis is shown in the section “Clinical words and terms to describe the SCS criteria” and Table C1 in the Appendix. The presence of the symptom was coded “yes.” Explicit denial of the symptom was coded “no.” Missing information about the symptom was coded “not reported.”

Our main outcome variable is a dichotomous proxy variable where we excluded entrapment and social withdrawal due to the high proportion of “not reported” for these symptoms. Patients were grouped according to high or low level of SCS symptoms. Then, 72 patients had a high level of symptoms and 638 had low level. For robustness and comparison, a second proxy variable was constructed; see the section “Alternative proxy variable” and Table B1 of the Appendix.

### Measures: Explanatory variables

To assess relevant group differences according to high or low level of SCS symptoms, we included traditional risk factors for suicide, PANSS-EC total score, clinical variables in relation to suicide risk, and demographic variables. Further details on definitions for the explanatory variables are listed in the section “List of variables” in the Appendix. See also earlier publications from the AA study and GAP study for details [20, 22].

### Statistical analyses

All statistical analyses were performed using the R programming language for statistical computing [33]. The main outcome variable was the dichotomized SCS high/low level. All explanatory variables were assessed one at a time for association with the outcome variable using logistic regression. Univariate logistic regression was used to give an estimated OR, 95% CI for the OR, and p-value [34]. Adjusted OR, adjusting by study AA/GAP (with CIs and p-value), was constructed by performing logistic regression with the study as an additional independent variable. For our categorical diagnosis variable, the effect of diagnosis was assessed by fitting two logistic regression models (one with the diagnosis variable and one without it), and the two models were compared using the likelihood ratio test. We control the FDR using the Benjamini–Hochberg procedure [32], and FDR-adjusted p-values are reported for each outcome variable analysis. We use a cut-off of 0.05 on the FDR-adjusted p-value to declare significance. If an FDR-adjusted p-value is 0.05, it means that if this is used as a cut-off for declaring significance, we would, on average, assume that 5% of the rejected hypotheses could be false positive results.

### Ethics

As recommended in The Declaration of Helsinki – Ethical Principles for Medical Research Involving Human Subjects, all participants who consented to participate in the study were assessed as able to provide informed consent by a specialist in psychiatry or clinical psychology. The Regional Ethical Committee approved the studies 2011/137 and 9565) and also approved the merging of the two studies (REK-nr 2014/1751). The studies are registered in ClinicalTrials.gov (ref. NCT01415323).

## Results

The sample consisted of 710 individuals. There were 363 women and 347 men. The age range was 18–84, with a mean age of 38.4. Table 1 shows the distribution of SCS symptoms described in the 710 EMRs through retrospective text analysis. There were few reports on entrapment and social withdrawal.

**Table 1.** Distribution of SCS symptoms described in the electronic medical records of 710 acutely admitted patients by retrospective text analysis

	Yes	No	Not reported
Entrapment	17	16	677
Social withdrawal	69	12	629
Disturbance in arousal	105	531	74
Affective dysregulation	83	567	60
Loss of cognitive control	110	529	71

Table 2 shows the distribution of the number of SCS symptoms described in EMR from 710 acutely admitted patients.

**Table 2.** Number of SCS symptoms described in the electronic medical records of 710 acutely admitted patients by retrospective text analysis

SCS symptoms described: 0	SCS symptoms described: 1	SCS symptoms described: 2	SCS symptoms described: 3	SCS symptoms described: 4	SCS symptoms described: 5
444/710	177/710	62/710	25/710	2/710	0/710

**Figure 1.**
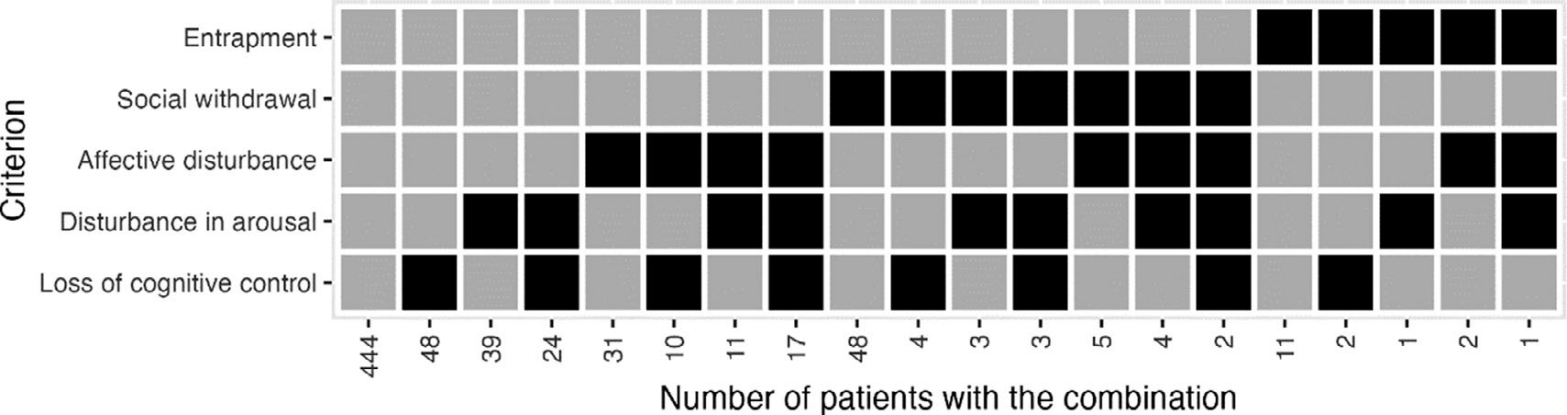
Combination of SCS symptoms described in the electronic medical records of 710 acutely admitted patients by retrospective text analysis.

Figure 1 shows the distribution and combination of SCS symptoms met in our sample. Gray squares are “no” or “not reported”. Black squares are “yes”. The first column shows that for 444 individuals, none of the symptoms were reported as “yes”. The second column shows that 48 individuals reported “yes” for loss of cognitive control only. The third column shows that 39 individuals reported “yes” for disturbance in arousal only. The fourth column shows that 24 individuals reported “yes” for both loss of cognitive control and disturbance in arousal, and so on.

Table 3 shows an overview of our explanatory variables, where the information is given for participants grouped in high- and low level of SCS symptoms. For all explanatory variables, we list the number and percentage of missing data. For the continuous explanatory variables (age, duration of stay and PANSS-EC total score), we give the median and first and third quartile for the high- and low-level groups. We also present tests for group difference, unadjusted and adjusted OR estimate with 95% CI and p-value (with two significant digits). The OR for age is related to an increase of 5 years, for the duration of stay of 1 day and for PANSS-EC total score of one unit. For the binary explanatory variables (all except age, duration of stay, PANSS-EC total score and diagnosis) we give the number of patients with the value listed (prior admittance equal to yes etc.) for the high- and low-level SCS group, together with the percentage of patients in each group.

**Table 3.** Overview of explanatory variables, where participants are grouped in high and low levels of symptoms of the suicidal crisis syndrome

	SCS high level *n* = 72	Missing	SCS low level *n* = 638	Missing	Unadjusted OR (95%CI), p	Adjusted OR (95%CI), p	FDR-adjusted p-value
Age, years	39.5 (29, 55)	0 (0%)	36.0 (25, 48)	0 (0%)	1.1 (0.99, 1.1), p = 0.095	1.1 (0.99, 1.1), p = 0.1	0.2
Women (%)	37 (51.4%)	0 (0%)	326 (51.1%)	0 (0%)	1.0 (0.62, 1.7), p = 0.96	1.0 (0.63, 1.7), p = 0.9	0.95
AA/GAP study	44/28	0 (0%)	336/302	0 (0%)	0.71 (0.43, 1.2), p = 0.17	0.71 (0.43, 1.2), p = 0.17	0.31
Duration of stay, days	8.0 (4, 14)	0 (0%)	6.0 (4, 10)	0 (0%)	1.20 (0.9988, 1.05), p = 0.051	1.02 (0.997, 1.04), p = 0.064	0.17
Prior admittance yes (%)	46 (63.9%)	0 (0%)	425 (66.6%)	2 (0.3%)	0.88 (0.53, 1.5), p = 0.62	0.89 (0.54, 1.5), p = 0.64	0.81
Involuntary referral	30 (41.7%)	0 (0%)	102 (16.0%)	0 (0%)	3.8 (2.2, 6.3), p = 4.6 × 10^−7^	3.6 (2.2, 6.1), p = 1.1 × 10^−6^	**6.4 × 10**^**−6**^
Involuntary admittance	23 (31.9%)	2 (2.8%)	57 (8.9%)	9 (1.4%)	4.9 (2.8, 8.6), p = 4.0 × 10^−8^	4.8 (2.7, 8.4), p = 9.1 × 10^−8^	**8.2 × 10**^**−7**^
Suicidality relevant for referral yes (%)	41 (56.9%)	4 (5.6%)	464 (72.7%)	11 (1.7%)	0.53 (0.32, 0.9), p = 0.017	0.52 (0.31, 0.89), p = 0.015	**0.044**
Intake suicide assessment, high risk yes (%)	28 (38.9%)	9 (12.5%)	149 (23.4%)	40 (6.3%)	2.4 (1.4, 4.1), p = 0.0011	2.4 (1.4, 4.2), p = 0.00098	**0.0035**
Suicidal thoughts, recent yes (%)	28 (38.9%)	10 (13.9%)	443 (69.4%)	27 (4.2%)	0.31 (0.18, 0.53), p = 1.7 × 10^−5^	0.31 (0.18, 0.53), p = 1.9 × 10^−5^	**8.6 × 10**^**−5**^
Suicide attempt, recent yes (%)	9 (12.5%)	25 (34.7%)	109 (17.1%)	126 (19.7%)	0.88 (0.39, 1.8), p = 0.73	0.85 (0.37, 1.8), p = 0.68	0.81
Insomnia, recent yes (%)	35 (48.6%)	30 (41.7%)	283 (44.4%)	273 (42.8%)	1.4 (0.66, 3.7), p = 0.39	1.4 (0.65, 3.7), p = 0.4	0.6
Substance abuse, recent yes (%)	25 (34.7%)	24 (33.3%)	210 (32.9%)	101 (15.8%)	1.7 (0.94, 3.1), p = 0.082	1.7 (0.93, 3.1), p = 0.083	0.19
Broken relationship	9 (12.5%)	57 (79.2%)	102 (16.0%)	466 (73.0%)	1.0 (0.36, 3.2), p = 0.96	1.0 (0.31, 3.6), p = 0.95	0.95
Lack of network	8 (11.1%)	57 (79.2%)	125 (19.6%)	396 (62.1%)	1.1 (0.37, 3.1), p = 0.9	1.1 (0.39, 3.4), p = 0.81	0.91
Loss of self esteem	5 (6.9%)	63 (87.5%)	42 (6.6%)	560 (87.8%)	1.1 (0.26, 4.6), p = 0.92	1.4 (0.32, 6.2), p = 0.67	0.81
PANSS-EC total score	14 (9, 18)	3 (4.2%)	8 (6, 12)	22 (3.4%)	1.2 (1.1, 1.2), p = 1.1 × 10^−13^	1.2 (1.1, 1.2), p = 4.6 × 10^−13^	**8.3 × 10**^−**12**^
Diagnosis (%)		0 (0%)		0 (0%)	P = 0.37	*P* = 0.39	0.6

The unadjusted and adjusted OR estimate and 95% CI and p-values are listed in the rightmost columns, with two significant digits (except for the duration of stay, where three significant digits are used). For the categorical variable diagnosis, there were no missing values, and only the p-value (for assessing if at least two categories have different effects from each other) and FDR-adjusted p-value are given in the two rightmost columns.

Significant differences between the high- and low SCS-level groups (OR different from 1) were found for suicidality relevant for referral, intake suicide assessment, recent suicidal thoughts, involuntary referral, involuntary admittance, and PANSS-EC total score.

The patients in the high-level group were less often referred due to suicidality (OR 0.53, FDR-adjusted p = 0.044). They were more often assessed with high risk for near-term suicidality at the intake assessment (OR 2.4, FDR-adjusted p = 0.0035). They reported less suicidal thoughts recent than the low-level group (OR 0.31, FDR-adjusted p = 8.6 × 10^−5^). We found significant differences between the high-level and low-level groups in the distribution of involuntary referral (OR 3.8, FDR-adjusted p < 6.4 × 10^−6^) and involuntary admittance (OR 4.8, FDR-adjusted p = 8.2 × 10^−7^). Group differences in PANSS-EC total score were highly significant with OR 1.2, FDR-adjusted p = 8.3 × 10^−12^. There were no significant differences in the distribution of diagnosis between the high- and low-level groups.

## Discussion

In this study, we compared two patient groups based on the occurrence of SCS symptoms in a sample of 710 individuals admitted to a Norwegian acute psychiatric department. First, we wanted to explore group differences between participants with high and low levels of SCS symptoms. Second, we wanted to see if there were any associations between the participants’ level of SCS symptoms and level of agitation, as measured by the PANSS-EC.

We used a manualized text analysis of the EMR to find information on SCS symptoms retrospectively. Based on this analysis [29], no participants in our sample had a description of all five SCS components. The SCS was first described in 2017 by Galynker and was only sparsely known when the study sample was admitted. This lack of knowledge could possibly lead to few reports of symptoms in the EMR, especially entrapment and social withdrawal, which have to be asked specifically about. This makes it difficult to conclude, based on the text analysis of EMR, that only part of the criteria was present in our study sample at admission. Today, it would have been possible to use the validated 61-item questionnaire SCI-2 to assess the prevalence of SCS symptoms at admission [19]. However, a recent review questions how practical the administration of such a measure is in certain clinical settings and further discusses the problem of overestimating the prevalence of psychopathological syndromes based on self-report [14]. The authors suggest further studies in which the presence of the SCS is determined using short clinician-rated measures, such as the SCS-Checklist (SCS-C) [35] or the abbreviated SCS-C [36].

Different ways of grouping SCS versus non-SCS according to a number of criteria met are tested in multiple studies [12, 13, 35]. In an article by Yaseen et al., the syndrome with all five criteria met had the best predictive value for SB within 4 weeks [12]. There have also been tested models with three and one criteria met, where models with three criteria were superior to those with one criterion in predicting SB [35]. In keeping with these findings, we made a proxy variable for high and low levels of SCS symptoms. With this proxy variable, we found approximately 10% of participants with high levels of SCS symptoms in our sample of acutely admitted patients. In a study who examined the occurrence of self-reported SCS symptoms in non-patient groups in 10 countries worldwide, Rogers et al. found a prevalence of SCS in 10% of the population [21].

We found that patients with high levels of SCS symptoms were more often assessed with a high risk for suicide at intake, more often referred and admitted involuntarily, and got a higher total score on PANSS-EC at admission. All of these factors are connected to more severe illness and less capability to take care of themselves at admittance.

The suicide risk assessment was done at admission and based on national guidelines for suicide risk assessment given by national health authorities [31] and local procedures [29]. This assessment consists of simple unstructured questions with no validated measures. Previous research from our department has shown that patients assessed with high risk for imminent suicide at intake, based on these procedures, have an 8.7 times higher OR of suicide within 3 years after discharge, compared to patients assessed with low risk for imminent suicide [29].

A more surprising result was that the high-level group reported less SI the last month before admission than the low-level group. Information about SI and SB was based on self-assessment. This is considered unreliably and has led to the development of SCS, among others, attempting to describe suicide risk without relying on the patients’ self-report of SI and SB. Usually, we consider that high level of SI is a sign of a more severe condition with a higher suicide risk [37]. Nevertheless, some studies have shown that SB can occur suddenly and often without much planning. Høyen et al. found that 50% of patients admitted to an acute psychiatric ward withheld some information on SI during admission when asked at discharge [5]. Not all patients experience SI when asked, and Deisenhammer found that SI could arise as shortly as 10 minutes preceding SB [6]. A prospective cohort study from a Norwegian sample of 1846 patients diagnosed with uni- or bipolar depressive episodes found that SI was not a predictor of completed suicide [38]. Another study by Fredriksen et al. found that patients with psychotic depression had a reduced ability to identify and communicate SI [39].

We found significant associations between agitation, as measured by the PANSS-EC total score, and our proxy variable SCS level. Individuals in the high-level group had a mean PANSS-EC total score of 14, compared to 8 in the low-level group (FDR-adjusted p = 8.3 × 10^−12^). Agitation is shown by Rogers et al. to be a risk factor for both suicide attempts and completed suicide [23]. Chu et al. [40] also found elevated levels of agitation to be a risk factor for acute suicide risk. Agitation is relatively easy to measure and observe at admission, and could probably give useful information in assessing acute risk for SB.

### Strengths and limitations

Our sample is relatively large in this field of research, with 710 consenting adults from clinical practice in Norway. Very few studies of SCS are conducted in large clinical samples outside the United States, and studies exploring the occurrence of SCS symptoms in clinical samples are sparse. When the patients were included, the concept of SCS as it is described today was sparsely known, and validated instruments such as the SCI-2 questionnaire or clinician-rated measures, including the SCS-C, did not exist. Our information about the SCS symptoms were based on symptom descriptions matching the criteria, collected by a retrospective text analysis of EMR. A retrospective, indirect study of a phenomenon like the present has, of course, several disadvantages, and further studies should be done by direct interview and observation of patients from similar clinical samples.

None of the patients in this sample met all five SCS criteria. This might be due to the lack of education on these symptoms when the patients were admitted, which in turn might have led to few reports in the EMRs. It is also possible that the text analysis used to find descriptions of these symptoms did not apply to the EMRs in retrospect and would have found more criteria if used at admission. This makes it difficult to conclude that the full SCS did not occur in our sample. We did find some of the criteria, which may point to a partial validation of the SCS.

The fact that we tested two different proxy variables and found the same group differences strengthens our analyses. The strong associations between our proxy variable and PANSS-EC total score also support the use of SCS symptoms as important individual criteria. Our selected variables for comparing participants with high and low levels of SCS symptoms were based on traditional risk factors for SB, demographic variables, and validated tools such as PANSS-EC. There is no international consensus as to which factors are most important, and different studies use different variables according to their knowledge. This can make it difficult to generalize and compare findings.

### Further research

Melzer et al. [14] conducted a review article of the literature on SCS from 2017 to 2022. There were no studies examining the occurrence of SCS; all studies so far were done in small patient samples, which could lead to the interpretation that the syndrome is relatively rare. They also found that almost all existing studies were conducted in the United States. It would be important to test translated versions of the SCI-2 questionnaire or clinician-rated measures, such as the SCS-C, in samples of high-risk patients outside the United States. It would also be important to investigate if there are factors and possible intervention points, such as medications or diagnoses, preceding the development of SCS. Our finding of the association between a high level of SCS symptoms and a higher PANSS-EC total score would also be interesting to examine further in prospective studies.

## Conclusions

We compared two groups with high and low levels of SCS symptoms and found that participants with high levels of SCS symptoms were more often assessed with high risk for suicide at the intake assessment, reported less SI last month before admission, were more often referred and admitted involuntarily, and were assessed as more agitated with PANSS-EC. These results indicate that an absence of expressed or acknowledged SI does not mean that the risk of suicide should be considered low. Unfortunately, the descriptions of SCS symptoms and, especially entrapment and social withdrawal, were sparse in the EMRs, possibly due to the lack of education on these symptoms at the time of enrollment. In clinical practice and further research, a thorough assessment of the patients, including SCS symptoms and level of agitation, should be collected.

## Data Availability

Data will be available on reasonable request.

## Trial registration

The Regional Ethical committee approved the studies (2011/137 and 9565) and also approved the merging of the two studies (REK-nr 2014/1751). The studies are registered in ClinicalTrials.gov (ref. NCT01415323).

## Appendix: Assessment of sample pooling

Table A1 shows the comparison of the samples from the AA and GAP studies. For all variables, we list the number and percentage of missing data. For the continuous variables (age, duration of stay, and PANSS-EC total score), we give the median and first and third quartile for the two samples together with the (unadjusted) OR estimate (comparing AA to GAP) with 95% CI and p-value (with two significant digits). The OR for age is related to an increase of 5 years, for the duration of stay of 1 day and a PANSS-EC total score of one unit. For the binary explanatory variables (all except age, duration of stay, PANSS-EC total score, and diagnosis), we give the number of patients with the value listed (prior admittance equal to yes, etc.) for the two samples, together with the percentage of patients in each sample. The unadjusted OR estimate and 95% CI and p-values are listed in the rightmost columns, with two significant digits. For the categorical variable diagnosis, the numbers are given for each of the diagnosis groups according to the International Classification of Diseases-10 (ICD-10). There were no missing values for this variable, and only the p-value (for assessing if at least two categories have different effects from each other) and FDR-adjusted p-value are given in the two rightmost columns.

We found significant group differences in the variables suicide attempt recent, involuntary referral, involuntary admittance, broken relationship, lack of network, loss of self-esteem, PANSS-EC total score, and diagnosis. Due to this finding, we decided to calculate both unadjusted and adjusted ORs in our analyses of the pooled sample to control for which study the participants belonged to.

**Table A1.** Comparison of the two samples from the AA and GAP studies before sample pooling

	AA (*n* = 380)	Missing	GAP (*n* = 330)	Missing	OR (95%CI), p	FDR-adjusted p-value
Age, years	37.5 (26, 48)	0 (0%)	34.0 (24, 49)	0 (0%)	0.05 (0, 0.12) p = 0.2224	0.45
Duration of stay, days	6. 0 (3,11)	0 (0%)	6.0 (4, 10)	0 (0%)	0.99 (0.97, 1.00) p = 0.15	0.21
Gender: Women (%)	184 (48.4%)	0 (0%)	179 (54.2%)	0 (0%)	1.26 (0.93, 1.72), p = 0.1324	0.2
Prior admittance (%)	249 (65.5%)	0 (0%)	222 (67.3%)	2 (0.6%)	1.1 (0.8, 1.5), p = 0.5764	0.54
Suicidality relevant for referral (%)	273 (71.8%)	14 (3.7%)	232 (70.3%)	1 (0.3%)	0.8 (0.6, 1.1), p = 0.2340	0.31
Intake suicide assessment, high risk yes (%)	86 (22.6%)	36 (9.5%)	91 (27.6%)	13 (3.9%)	1.2 (0.8, 1.7), p = 0.2925	0.33
Suicidal thoughts, recent yes (%)	234 (61.6%)	36 (9.5%)	237 (71.8%)	1 (0.3%)	1.2 (0.9, 1.7), p = 0.2745	0.33
Suicide attempt, recent yes (%)	71 (18.7%)	134 (35.3%)	47 (14.2%)	17 (5.2%)	0.4 (0.3, 0.7), **p = 0.00096**	**0.0012**
Insomnia, recent yes (%)	192 (50.5%)	130 (34.2%)	126 (38.2%)	173 (52.4%)	1.2 (0.7, 2.0), p = 0.4606	0.44
Substance abuse, recent yes (%)	127 (33.4%)	84 (22.1%)	108 (32.7%)	41 (12.4%)	0.8 (0.6, 1.1), p = 0.1781	0.24
Involuntary referral yes (%)	88 (23.2%)	0 (0%)	44 (13.3%)	0 (0%)	0.5 (0.3, 0.8), **p = 0.00096**	**0.005**
Involuntary admittance yes (%)	54 (14.2%)	11 (2.89%)	26 (7.9%)	0 (0%)	0.5 (0.3, 0.8), **p = 0.006**	**0.023**
Broken relationship	65 (17.1%)	305 (80.26%)	46 (13.9%)	218 (66.1%)	0.1 (0, 0.2), **p = 0.0000000019**	**2.9 × 10**^−7^
Lack of network	66 (17.4%)	278 (73.16%)	67 (20.3%)	175 (53.0%)	0.4 (0.2, 0.7), **p = 0.0009**	**0.005**
Loss of self esteem	24 (6.3%)	348 (91.58%)	23 (7.0%)	275 (83.3%)	0.2 (0.1, 0.6), **p = 0.016**	**0.017**
SCS high level	57 (15%)		43 (13%)		0.8 (0.5, 1.3), p = 0.52	0.59
PANSS-EC total score	9 (7, 13)	11 (2.9%)	8 (6,11)	14 (4.2%)	0.94 (0.91, 0.97), p = 0.00041	**0.0039**
Diagnosis (%)		0 (0%)		0 (0%)	0.1, **p = 0.01561**	**0.044**
Behavioral and emotional disorders with onset usually occurring in childhood and adolescence	4 (1.1%)		3 (0.9%)		0.9 (0.2, 4.0), p = 1	0.12
Behavioral syndromes associated with physiological disturbances and physical factors	6 (1.6%)		1 (0.3%)		0.2 (0.0, 1.4), p = 0.13	0.16
Disorders of adult personality and behavior	34 (8.9%)		35 (10.6%)		1.2 (0.7, 2.0), p = 0.53	0.1
Disorders of psychological development	4 (1.1%)		6 (1.8%)		1.7 (0.5, 6.6), p = 0.53	0.11
Mental and behavioral disorders due to psychoactive substance use	80 (21.1%)		55 (16.7%)		0.8 (0.5, 1.1), p = 0.15	0.33
Mental retardation	3 (0.8%)		2 (0.6%)		0.8 (0.1, 5.0), p = 1	0.12
Mood (affective) disorders	133 (35.0%)		126 (38.2%)		1.1 (0.8, 1.6), p = 0.39	0.1
Neurotic, stress-related and somatoform disorders	34 (8.9%)		42 (12.7%)		1.5 (0.9, 2.4), p = 0.11	0.06
Organic, including symptomatic, mental disorders	18 (4.7%)		7 (2.1%)		0.4 (0.2, 1.1), p = 0.067	0.21
Schizophrenia, schizotypal and delusional disorders	51 (13.4%)		28 (8.5%)		0.6 (0.4, 1.0), p = 0.042	0.33
Uncertain mental disorder	13 (3.4%)		25 (7.6%)		2.3 (1.2, 4.7), p = 0.018	0.053

Significant p-values (below 0.05) are highlighted with bold typing

### Alternative SCS proxy variable

In our second proxy variable, we included all five SCS criteria and defined the high-level group as participants with a “yes” for the A criterion and/or “yes” for at least two of the five B-criteria. When we analyzed the second proxy variable, we obtained similar results to those for the first proxy variable. Differences included a significant group difference in duration of stay, and suicidality relevant for referral was not significantly different between high- and low-level groups. The other results were essentially unchanged (see Table B1).

**Table B1.** Overview of our explanatory variables. Participants split in high- and low level suicidal crisis syndrome using the proxy 2 definition

	High-level SCS	Missing	Low-level SCS	Missing	Unadjusted OR (95%CI), p	Adjusted OR (95%CI), p	FDR-adjusted p-value
Age, years	38.5 (26, 51)	0 (0%)	36.0 (25, 48)	0 (0%)	1.0 (0.98, 1.1), p = 0.17	1.0 (0.98, 1.1), p = 0.17	0.31
Duration of stay, days	7.5 (4, 13)	0 (0%)	6.0 (4, 10)	0 (0%)	1.03 (1.01, 1.05), p = 0.0067	1.03 (1.01, 1.05), p = 0.0078	**0.024**
From study (AA/GAP)	57/43	0 (0%)	323/287	0 (0%)	0.85 (0.55, 1.3), p = 0.45	0.85 (0.55, 1.3), p = 0.45	0.58
Gender: Women (%)	48 (48%)	0 (0%)	315 (51.6%)	0 (0%)	0.86 (0.57, 1.3), p = 0.5	0.87 (0.57, 1.3), p = 0.53	0.35
Prior admittance yes (%)	61 (61%)	0 (0%)	410 (67.2%)	2 (0.3%)	0.76 (0.49, 1.2), p = 0.21	0.76 (0.49, 1.2), p = 0.21	0.63
Suicidality relevant for referral yes (%)	62 (62%)	4 (4%)	443 (72.6%)	11 (1.8%)	0.64 (0.41, 1.0), p = 0.057	0.64 (0.41, 1.0), p = 0.054	0.14
Intake suicide assessment, high risk yes (%)	39 (39%)	10 (10%)	138 (22.6%)	39 (6.4%)	2.4 (1.5, 3.8), p = 0.00019	2.4 (1.5, 3.8), p = 0.00017	**0.00078**
Suicidal thoughts, recent yes (%)	51 (51%)	11 (11%)	420 (68.9%)	26 (4.3%)	0.52 (0.33, 0.83), p = 0.0056	0.52 (0.33, 0.83), p = 0.0056	**0.02**
Suicide attempt, recent yes (%)	15 (15%)	30 (30%)	103 (16.9%)	121 (19.8%)	1.0 (0.54, 1.8), p = 0.94	1.0 (0.54, 1.9), p = 0.92	0.92
Insomnia, recent yes (%)	47 (47%)	44 (44%)	271 (44.4%)	259 (42.5%)	1.5 (0.76, 3.5), p = 0.26	1.5 (0.75, 3.5), p = 0.27	0.4
Substance abuse, recent yes (%)	34 (34%)	29 (29%)	201 (33%)	96 (15.7%)	1.4 (0.87, 2.4), p = 0.16	1.4 (0.87, 2.4), p = 0.15	0.3
Involuntary referral (%)	34 (34%)	0 (0%)	98 (16.1%)	0 (0%)	2.7 (1.7, 4.3), p = 3.2 × 10^−5^	2.7 (1.7, 4.3), p = 4.5 × 10^−5^	**0.00027**
Involuntary admittance (%)	26 (26%)	2 (2%)	54 (8.9%)	9 (1.4%)	3.7 (2.1, 6.2), p = 1.5 × 10^−6^	3.6 (2.1, 6.2), p = 2.1 × 10^−6^	**1.9 × 10**^−**5**^
Broken relationship	14 (14%)	75 (75%)	97 (15.9%)	448 (73.4%)	0.85 (0.37, 2.0), p = 0.71	0.92 (0.35, 2.4), p = 0.86	0.91
Lack of network	16 (16%)	73 (73%)	117 (19.2%)	380 (62.3%)	1.4 (0.63, 3.2), p = 0.41	1.5 (0.67, 3.6), p = 0.32	0.44
Loss of self esteem	9 (9%)	84 (84%)	38 (6.2%)	539 (88.4%)	1.1 (0.38, 3.4), p = 0.84	1.4 (0.44, 4.6), p = 0.57	0.64
PANSS-EC total score	12 (8, 17)	4 (4.0%)	8 (6, 12)	21 (3.4%)	1.1 (1.1, 1.2), p = 1.1 × 10^−11^	1.1 (1.1, 1.2), p = 2.0 × 10^−11^	**3.6 × 10**^−10^
Diagnoses (%)		0 (0%)		0 (0%)	0.11	0.15	0.3
Behavioral and emotional disorders with onset usually occurring in childhood and adolescence	0		7				
Behavioral syndromes associated with physiological disturbances and physical factors	2		5				
Disorders of adult personality and behavior	6		63				
Disorders of psychological development	0		10				
Mental and behavioral disorders due to psychoactive substance use	19		116				
Mental retardation	0		5				
Mood (affective) disorders	40		219				
Neurotic, stress-related and somatoform disorders	7		69				
Organic, including symptomatic, mental disorders	4		21				
Schizophrenia, schizotypal and delusional disorders	17		62				
Uncertain mental disorder	5		33				

Significant p-values are highlighted with bold typing

### Clinical words and terms to describe the SCS criteria

Table C1 shows the clinical words and terms used to describe the SCS criteria in our manualized procedure. The words are listed in English and Norwegian.

**Table C1.** Clinical words and terms used to describe the SCS criteria, in English and Norwegian

	English	Norwegian
A Criterion	Entrapment	Fanget
	Unbearable situation	Uutholdelig livssituasjon
	Feeling of being trapped	Ingen utvei
	Hopelessness/despondent	Vilt fortvilet over egen situasjon
	Extreme desperation	Ekstrem desperasjon
	Inability to escape an intolerable situation	Uten mulighet for å unnslippe
B Criteria	Disturbance in arousal	Ytre motorisk uro
	Restlessness	Motorisk uro/rastløshet
	High paranoia readiness	Høy paranoid beredskap
	Hypervigilance	Oppfarende/vaktsom
	Agitated	Agitert
	Irritability	Irritabel
	Tense	Forknytt/anspent
	Affective component	Affektiv komponent
	Suffering	Lidende
	Desperation	Desperasjon
	Internal pressure	Indre press
	Anger	Sinne
	Mental/emotional pain	Psykisk smerte
	Extreme anxiety	Ekstrem angst
	Acute anhedonia	Akutt anhedoni
	Cognitive component	Kognitiv komponent
	Psychomotor restlessness	Psykomotorisk uro
	An overwhelming profusion of negative thoughts	Tankepress
	Racing thoughts	Tankekjør/tankemyldring
	Suppresion of thoughts	Tankeundertrykkelse
	Rumination	Ruminering
	Repetitive	Repeterende
	Irrational	Irrasjonell
	Fragmented	Fragmentert
	Mental tension	
	Confused	Forvirret/oppløst
	Mentally restless	Mentalt urolig
	Cognitive overload	Tankeflom/tankekaos
	Cognitive rigidity	Rigiditet/fastlåst

### List of variables

#### Outcome variable

Our main outcome variable is the first proxy variable, where we excluded entrapment and social withdrawal. We performed the same analyses with the second proxy variable with all five SCS criteria included as a control analysis.

#### Explanatory variables

To assess relevant group differences in the outcome variable, we included age, gender, duration of stay, suicidal thoughts recent, suicide attempt recent, insomnia recent, substance abuse recent, prior admittance, intake suicide assessment, referral paragraph, specialist-assessed paragraph within 24 hours of admittance, PANSS-EC total score and diagnosis.

**Suicidal thoughts recently** (last month) are addressed at every admission to the acute ward. This is self-reported information. Although some studies have shown that as many as 50–75% of patients who committed suicide [4, 5], denied suicidal thoughts when last asked, it is also shown to be an important predictor of suicide [41] and considered an important part of the suicide risk evaluation.

**Previous suicide attempt(s)** is probably one of the strongest predictors/risk factors for new suicide attempts. A study conducted by Berman [1] found that among suicide victims, 45% had a history of a previous attempt, almost half of them within 30 days of their death. We included self-reported suicide attempt(s) recently (within 30 days) as an explanatory variable in our study.

**Insomnia.** Pigeon et al. [42] conducted a meta-analysis and found an association between sleep disturbances and suicidal thoughts and behavior. The association was not affected by depression, which could be a potential confounder. Bernert et al. [43] reviewed existing literature and found sleep disturbances to be a potential empirical risk factor for SB.

**Substance abuse.** Yoshimasu et al. [44] conducted a meta-analysis of 16 psychological autopsy studies with a case–control design and found that substance-related disorders were strongly associated with suicidal risk. Information about self-reported substance abuse last month before admission was gathered from EPJ.

**Relational issues**. Stone et al. [45] found that 42% of suicide victims in the United States had relationship problems. Information regarding loss of self-esteem, broken relationships, and lack of network was gathered from EPJ.

**Intake suicide assessment** by the doctor on duty. It consisted of questions about suicidal thoughts and/or behavior last month and lifetime and the other traditional risk factors mentioned above. It also included clinical state variables such as anxiety, agitation, hopelessness, and so forth. The doctor on duty discussed the evaluation with the responsible psychiatrist and decided “high-risk” or “low-risk” for suicide.

**Involuntary referral and involuntary admittance** was assessed as described by Wynn et al. [46]. Patients were referred to the hospital either voluntarily or by coercion. At admission, some of the patients referred by coercion were admitted voluntarily. Information about involuntary referral and admittance were gathered from EPJ.

**Diagnosis.** Cavanagh et al. [47] found in their psychological autopsy study that 90% of suicide victims had a mental disorder. We have included the diagnosis given for the admittance since it describes the relevant condition[s) for the patient’s stay. We have used the ICD-10 [48].

**The PANSS-EC total score** was measured by the doctor on duty during the first 24 hours of admission to assess the patient’s level of agitation [28]. It consists of five items: excitement, tension, hostility, uncooperativeness, and poor impulse control. Each item is rated from 1 (not present) to 7 (extremely severe) and does not rely on self-report. Scores range from 5 to 35, and mean scores >20 clinically correspond to severe agitation. It is mostly used for grading psychotic symptoms, but given recent research on high-risk states such as psychotic depression [39], it may provide useful information in acute psychiatric settings as well.
